# Deciphering serous ovarian carcinoma histopathology and platinum response by convolutional neural networks

**DOI:** 10.1186/s12916-020-01684-w

**Published:** 2020-08-18

**Authors:** Kun-Hsing Yu, Vincent Hu, Feiran Wang, Ursula A. Matulonis, George L. Mutter, Jeffrey A. Golden, Isaac S. Kohane

**Affiliations:** 1 Department of Biomedical Informatics, Harvard Medical School, Boston, MA USA; 2 Department of Pathology, Brigham and Women’s Hospital, Boston, MA USA; 3 Department of Bioengineering, University of California San Diego, San Diego, CA USA; 4 Department of Electrical Engineering, Stanford University, Stanford, CA USA; 5 Division of Gynecologic Oncology, Dana-Farber Cancer Institute, Boston, MA USA

**Keywords:** Digital pathology, Platinum response, Gene expression, Proteomics, Machine learning, Serous ovarian carcinoma

## Abstract

**Background:**

Ovarian cancer causes 151,900 deaths per year worldwide. Treatment and prognosis are primarily determined by the histopathologic interpretation in combination with molecular diagnosis. However, the relationship between histopathology patterns and molecular alterations is not fully understood, and it is difficult to predict patients’ chemotherapy response using the known clinical and histological variables.

**Methods:**

We analyzed the whole-slide histopathology images, RNA-Seq, and proteomics data from 587 primary serous ovarian adenocarcinoma patients and developed a systematic algorithm to integrate histopathology and functional omics findings and to predict patients’ response to platinum-based chemotherapy.

**Results:**

Our convolutional neural networks identified the cancerous regions with areas under the receiver operating characteristic curve (AUCs) > 0.95 and classified tumor grade with AUCs > 0.80. Functional omics analysis revealed that expression levels of proteins participated in innate immune responses and catabolic pathways are associated with tumor grade. Quantitative histopathology analysis successfully stratified patients with different response to platinum-based chemotherapy (*P* = 0.003).

**Conclusions:**

These results indicated the potential clinical utility of quantitative histopathology evaluation in tumor cell detection and chemotherapy response prediction. The developed algorithm is easily extensible to other tumor types and treatment modalities.

## Background

Ovarian cancer is one of the deadliest cancers in women worldwide, causing 151,900 deaths per year [1]. The lifetime risk of a woman getting ovarian cancer is 1 in 78. Serous ovarian carcinoma is the most common type of ovarian cancer, accounting for 52% of all cases. Due to the non-specific symptoms in the early stages, over 79% of ovarian cancer patients are diagnosed at stage III or IV [2], when tumor cells have spread to retroperitoneal lymph nodes or distant organs [3], further contributing to the unfavorable prognoses of this deadly disease [2].

Histopathology evaluation is the gold standard for diagnosing ovarian cancer and identifying the histological types [4]. Interpretation of the cellular morphology defines the various ovarian cancer types and guides treatment planning [4]. Within the category of serous ovarian carcinoma, are two subtypes, designated as high grade and low grade, that differ in pathogenesis, histologic appearance, and clinical course [5]. There is molecular lineage continuity, histologic, and clinical data supporting fallopian tube origins for many high-grade tumors, which are more aggressive and are associated with shorter overall survival than low-grade serous cancers [5]. Grading of ovarian tumors is best performed by pathologists with expertise in ovarian tumors, but inter-observer variation in grading has been reported. For example, three independent studies have reported the reproducibility for grade classification as fair to moderate (*κ* = 0.25–0.58) [6–8]. This variation in histopathologic interpretation would contribute to inaccurate prognostic prediction, suboptimal treatments, and loss of quality of life [9].

Platinum-based chemotherapy is the standard treatment for patients with advanced stages of serous ovarian carcinoma [10, 11]. Platinum-based therapy is unfortunately associated with a wide range of adverse effects, including myelosuppression, immunosuppression, hearing loss, nephrotoxicity, and neurotoxicity, as well as nausea and vomiting [12]. In addition, the clinical response to platinum drugs varies across patients [13]. The platinum-free interval (PFI), defined as the interval between the completion of platinum-based chemotherapy and the clinical detection of tumor relapse, is used to quantify the chemotherapy response [14]. Currently, it is very difficult to predict if a patient will respond to platinum-based chemotherapy [15, 16]. While histopathology continues to play a central role in diagnosing and subtyping ovarian cancer [4], it is uncertain if any histopathologic patterns are associated with a better or worse PFI. A reproducible set of pathological features indicative of chemotherapy response would facilitate treatment selection for advanced-stage ovarian cancer patients [13].

With the recent advances in the reliability of whole-slide histopathology scanners and high-throughput omics profiling [17] coupled with innovative machine learning algorithms and computer vision techniques, it is now possible to discover the previously unknown associations between microscopic tumor cell morphology and molecular pathways. Machine learning models have shown great promise in associating histopathology patterns with patients’ diagnoses and prognoses. Previously, studies have identified the correlations between quantitative morphological features and patient survival in breast cancer [18] and lung cancer [17, 19, 20]. Recent advances in convolutional neural networks employed multiple convolutional layers to extract high-level features from the images and used pooling layers to achieve translational invariance [21]. Such methods have attained human-level performance in diagnosing chest radiographs, fundus photographs, and photographs of skin lesions [21, 22]. Studies also showed that quantitative histopathology analyses can provide correlations between tumor tissue morphology and certain somatic variations related to prognoses [19]. These results indicate that high-resolution whole-slide pathology images contain underutilized biological signals of clinical importance. In addition, visualization approaches, such as the gradient-weighted class activation maps (grad-CAMs) [23], can facilitate the interpretation of machine learning models and identify the image regions associated with the outcomes of interest [21].

In this study, we developed convolutional neural network models to analyze cellular patterns and morphology in a series of patients with serous ovarian carcinoma. Our models successfully identified ovarian cancer cells, classified histology grade and transcriptomic subtypes, and predicted patients’ response to platinum-based chemotherapy. We further conducted differential expression and enrichment analyses to connect findings from our quantitative histopathology studies with the underpinning molecular pathways. Importantly, our approaches are completely data-driven and can accommodate new categories of cancers or the response to other novel treatment strategies. The development of these prediction algorithms will contribute invaluable information to precision cancer care [17].

## Methods

### Acquisition of histopathology, transcriptomics, and proteomics data from ovarian cancer patients

Five hundred eighty-seven serous ovarian carcinoma patients participated in The Cancer Genome Atlas (TCGA) [24] were included in this study. Whole-slide histopathology scans, pathology reports, and RNA-sequencing data were acquired from the Genomic Data Commons [24]. Histopathology slides of the adjacent benign tissue were also obtained. These slides contain high-resolution, colored, and two-dimensional images stored in “.svs” file format. All 587 patients have digital whole-slide histopathology slides, and 305 of these patients have raw RNA-sequencing information. Proteomics data generated by the Clinical Proteomic Tumor Analysis Consortium (CPTAC) [25] of 174 patients were obtained from the CPTAC Data Coordinating Center.

Although the pathology samples were collected at different hospitals, all samples underwent multi-omics profiling passed the required quality control requirements (including having a board-certified pathologist to review the histologic sections of the top and bottom portions of the samples to confirm the diagnoses and to make sure that the samples contained an average of 70% tumor cell nuclei with less than 20% necrosis) [24]. RNA-seq of the ovarian cancer samples were performed by the Cancer Genomic Characterization Center (CGCC) at the University of North Carolina using the Illumina HiSeq platform. The short reads generated by the sequencers were processed by a standard alignment pipeline using the Spliced Transcripts Alignment to a Reference (STAR) software. HTSeq with gene annotations from GENCODE v22 was employed to compute the fragments per kilobase of transcript per million mapped reads (FPKM) for each gene in each sample [26]. Using the same experimental and bioinformatics procedures for all samples ensures that the resulting transcriptomics data are comparable. Proteomics data were generated by the CPTAC using LC-tandem mass spectrometry on an Orbitrap mass spectrometer. Peptide identification was conducted by database searching using the RefSeq human protein sequence database, and the identified peptides were assembled into proteins. Isobaric tags for relative and absolute quantitation (iTRAQ) reporter ion intensities were used for protein quantitation [25]. Transcriptomic subtypes were defined by the method proposed by TCGA Research Network, which was based on the microarray profiling results (*n* = 553) [27]. Four different subtypes, including differentiated, immunoreactive, mesenchymal, and proliferative subtypes, were identified. In addition to the subtype categories, the transcriptomic subtype scores were obtained for each sample from the TCGA publication [27].

### Convolutional neural networks for histopathology image classification

AlexNet [28], GoogLeNet [29], and VGGNet (16-layer configuration) [30] architectures were employed to build classification models to distinguish the histopathology slides with tumor cells from slides of adjacent benign tissue. These multi-layer artificial neural networks are very flexible and can easily overfit the training data, especially when the size of the training dataset is small. To reduce the risk of overfitting, pre-trained convolutional neural networks with weights from the ImageNet dataset were used as the baseline frameworks. Previous studies showed that these frameworks trained on millions of images can capture the crucial visual elements in the images, such as edges, circles, and object bulbs, which can serve as the building blocks for more complicated image recognition tasks [31, 32]. To establish specialized histopathology classification models, the weights of all neural connections in the networks were fine-tuned by the backpropagation algorithm [33], with the histopathology images as the input and the diagnostic labels (cancerous tissue versus adjacent benign tissue) in the training set as the output. The same transfer learning approaches were employed to predict the binarized histological grades (grades 1–2 versus grade 3) and to identify the four transcriptomic subtypes. Histological grades were binarized due to the known inter-rater variability in grade annotation [8].

The TCGA dataset was randomly divided into distinct training (80% of all cases: 973 malignant and 127 benign slides for the malignancy detection task; 396 high-grade and 60 low-to-moderate-grade patients for the grade classification task) and test sets (20%: 243 malignant and 32 benign slides for the malignancy detection task; 99 high-grade and 15 low-to-moderate-grade patients for the grade classification task), in order to evaluate the classification performance objectively. Patients without grade annotation (*n* = 16) or with anaplastic grade (*n* = 1) were excluded from the grade classification task. The models were developed using only the training set, and all hyperparameters (parameters that defined the neural network design and the model training process) were optimized by fivefold cross-validation on the training set. This approach ensured that there was no information leakage in our model training and optimization process. Thus, the test set performance served as an objective measure of the external validity of our models. The optimal baseline learning rate was 0.001 for GoogLeNet and AlexNet, 0.0005 for VGGNet. The optimal weight decay was 0.0005 for AlexNet, 0.0002 for GoogLeNet, and 0.0002 for VGGNet. The optimal momentum was 0.9 for all models, and L2 regularization was used in all convolutional neural network architectures. The final cancer detection and grade classification models were evaluated by the untouched test set. Fivefold cross-validation was used in transcriptomic subtype classification due to the smaller number of samples in the four subtypes. To ensure the reproducibility of the results, the training-test set partition and the model optimization processes were repeated three times for each classification task. The areas under the receiver operating characteristic curves were computed and compared.

Modern deep convolutional neural networks possess millions of parameters, making them very difficult to interpret. To better visualize and understand the behaviors of the trained neural network models, gradient-weighted class activation maps (grad-CAMs) [23] were employed to identify the relative importance of the regions in the input image in each classification task. The grad-CAMs algorithm visualized the impact of perturbations to the input pixels on the output class, thereby quantifying the relevance of each image region in the task [23]. The relative importance of each pixel was rescaled to a value between 0 and 1, and the “jet” colormap in the python Matplotlib library [34] was used to visualize the results. The grad-CAMs were generated using the keras-vis library in python.

### Predicting platinum-free interval using histopathology images

Platinum-free interval (PFI) is often used as a quantitative estimate of the efficacy of platinum-based chemotherapy [14]. It is defined as the time interval between the completion of chemotherapy treatment and the onset of tumor relapse [14]. To delineate the morphological patterns of tumor tissue associated with the platinum response, a subset of patients with PFI information was identified, and convolutional neural networks with a regression output were developed and applied to their histopathology images. Given its robust performance in the grade classification task, VGGNet was employed to build the regression models. The root-mean-square propagation algorithm (RMSProp) [35] with the mean squared error loss function was employed to train the machine learning models. Due to the limited number of cases with platinum response information (*n* = 277), fivefold cross-validation was used to evaluate the performance of the neural networks. This cross-validation design allowed each patient to serve in the test set once and ensured that the data from the same patients were not included in both the training and test sets simultaneously, which enabled objective evaluation of the model performance. The machine learning model computed a predicted response index for each patient, and the median index observed in the training set was used to divide the patients in the test set into an early-relapse group and a late-relapse group. The log-rank test was used to examine the differences in platinum response between the two predicted groups.

### Connecting histopathology patterns with transcriptomic and proteomic profiles

To connect the histopathology patterns with molecular aberrations, differential expression, enrichment, and pathway analyses were conducted using the transcriptomics and proteomics data to reveal the differences in functional omics between patients with different grades and response to platinum-based chemotherapy.

In the tumor grade analyses, the fold change of each gene and protein between the high-grade group and the low-to-moderate-grade group was computed. Exploratory analyses were conducted using the OASISPRO tool [36]. Since none of the proteins attained more than twofold changes between the grade groups, genes and proteins with fold changes in the 99th percentile or the 1st percentile were identified. The gene and protein expression differences in the two grade groups were tested by the Wilcoxon rank-sum test, with the *P* value corrected by the Benjamini-Hochberg procedure. Gene Ontology (GO) [37] and KEGG pathway [38] enrichment analyses were performed to characterize the biological functions and molecular pathways associated with the identified genes and proteins. The STRING tool [39] was employed to visualize the protein-protein interactions among the identified proteins, with color-coded edges showing the sources of the curated protein-protein interactions in the STRING database. To investigate the predictive values of proteomic and transcriptomic profiles for tumor grade, multi-layer neural networks were built to distinguish tumor grades using the omics data, and the prediction performance was evaluated by fivefold cross-validation. Using the training data of each fold, the optimal architecture of the neural networks were determined by a hyperparameter search with one to five hidden layers.

In the platinum response analyses, Spearman’s correlation coefficient between the PFI groups and the expression level of each gene or protein was calculated, and a correlation test was performed and corrected by the Benjamini-Hochberg procedure. GO and KEGG pathway analyses were conducted, and protein-protein interactions among the most relevant proteins were examined using the same tools. Similar multi-layer neural network approaches were employed to evaluate the predictive values of transcriptomics and proteomics data for the PFI groups. All statistical analyses were performed using R version 3.6.

## Results

### Patient characteristics

**Fig. 1:**
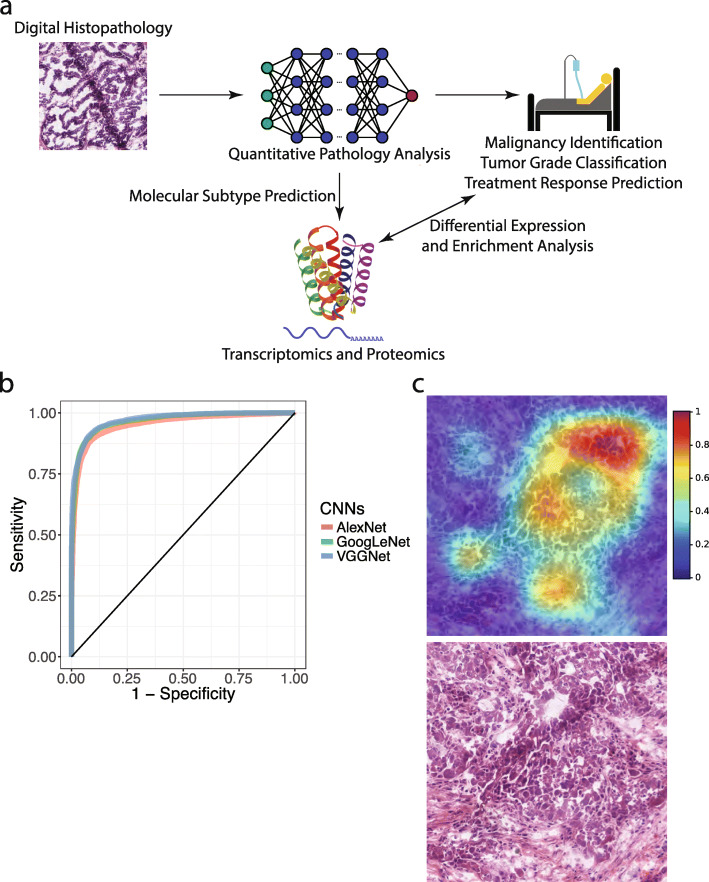
Integrative histopathology-functional omics analyses on serous ovarian carcinoma. **a** A model of the informatics workflow in this study. **b** Convolutional neural networks identified regions with tumor cells of serous ovarian carcinoma. Receiver operating characteristic (ROC) curves of convolutional neural networks that classified regions with tumor cells from those without tumor cells in the independent test set are shown. Areas under the receiver operating characteristic curves (AUCs) in the independent test set: AlexNet = 0.955 ± 0.010; GoogLeNet = 0.974 ± 0.004; VGGNet = 0.975 ± 0.001. **c** Gradient-weighted class activation maps (grad-CAMs) confirmed that the CNN models focused on the cancerous part of the histopathology slides when classifying malignant tissues from benign ones. The original hematoxylin-and-eosin-stained histopathology image was also shown

Five hundred eighty-seven ovarian cancer patients were identified from The Cancer Genome Atlas [24]. The majority of the patients had grade 2 or grade 3 diseases. Table 1 summarized the patient characteristics of our study cohort. Figure 1a shows the model of our data integration and analytics workflow. Overall, we developed histopathology-based machine learning models for cancer identification, grade classification, transcriptomic subtype recognition, and chemotherapy response prediction using digital whole-slide pathology images, and we employed proteomics and transcriptomics data of the same patients to link our quantitative pathology findings with the relevant molecular pathways.

**Table 1:** Clinical characteristics of serous ovarian carcinoma patients in this study

Clinical characteristics	Summary
Serous Ovarian Carcinoma Patients with Clinical Data	*N*=587
Number of Tumor Histopathology Image Series	1216
Number of Histopathology Image Series of Adjacent Benign Tissue	159
Age	59.74 ± 11.53 years
Race	
White	498 (84.84 %)
Black or African American	34 (5.79 %)
Asian	20 (3.41 %)
American Indian or Alaska Native	3 (0.51 %)
Native Hawaiian or Other Pacific Islander	1 (0.17 %)
Race Not Available	31 (5.28 %)
Anatomical subdivision of the tumor	
Bilateral	400 (68.14 %)
Left	82 (13.97 %)
Right	71 (12.10 %)
Not Available	34 (5.79 %)
Stage	
Stage I	17 (2.90 %)
Stage II	30 (5.11 %)
Stage III	446 (75.98 %)
Stage IV	89 (15.16 %)
Stage Not Available	5 (0.85 %)
Grade	
Grade 1	6 (1.02 %)
Grade 2	69 (11.75 %)
Grade 3	495 (84.33 %)
Grade 4	1 (0.17 %)
Grade Not Available	16 (2.73 %)
Transcriptomic subtypes	
Differentiated	140 (23.85 %)
Immunoreactive	159 (27.09 %)
Mesenchymal	104 (17.72 %)
Proliferative	150 (25.55 %)
Not Available	34 (5.79 %)

### Convolutional neural networks identified histopathology images with cancer cells

We used convolutional neural networks to detect histopathology images with cancer cells. Results showed that our method accurately identified the images with cancer cells from those without tumor, with area under the receiver operating characteristics curve (AUC) > 0.95 (Fig. 1b; AlexNet AUC = 0.955 ± 0.010; GoogLeNet AUC = 0.974 ± 0.004; VGGNet AUC = 0.975 ± 0.001). Grad-CAMs [23] confirmed that clusters of tumor cells received higher weights in differentiating malignant cells from the adjacent dense benign tissue (Fig. 1c). These results indicated that convolutional neural networks can distinguish regions with serous ovarian carcinoma from the unaffected regions of the ovaries.

### Convolutional neural networks predicted the histopathology grade of the patients

**Fig. 2:**
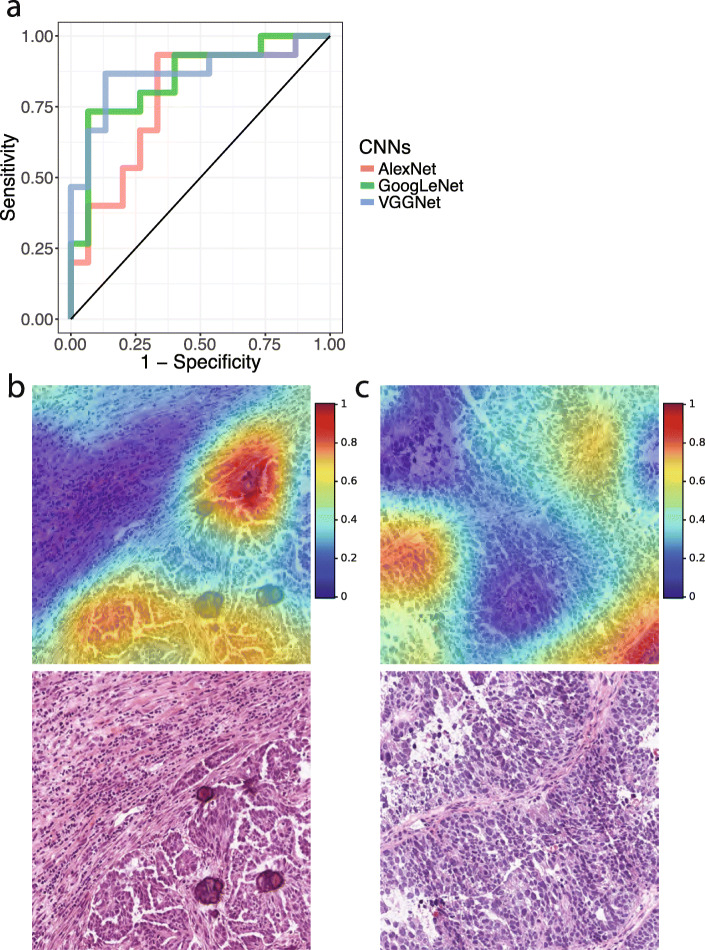
Quantitative histopathology analysis identified tumor grade. **a** ROC curves of convolutional neural networks that classified the pathology grade of serous ovarian carcinoma. The sensitivity and specificity for identifying high-grade serous ovarian carcinoma are shown. AUC in the independent test set: AlexNet = 0.760 ± 0.082; GoogLeNet = 0.810 ± 0.067; VGGNet = 0.812 ± 0.088. **b** The gradient-weighted class activation map (grad-CAM) of a histopathology image of a low-grade ovarian cancer patient and the original hematoxylin-and-eosin-stained histopathology image. Tumor cells and differentiated cellular structures received higher weighted in the grad-CAM. **c** The grad-CAM of a histopathology image of a high-grade ovarian cancer patient and the original hematoxylin-and-eosin-stained histopathology image. Clusters of tumor cells with poor differentiation were highlighted by the grad-CAM

Histopathology grade characterizes the differentiation level of serous ovarian adenocarcinoma tissue, and it is associated with the prognoses of ovarian cancer patients [5]. However, inter-rater variability in grade annotations has been reported [6–8]. To examine the utility of quantitative histopathology analysis on determining tumor grade, we employed convolutional neural networks to classify image patterns of patients with different histopathology grades. We employed the same transfer learning approaches with the same set of neural network architectures, but we retrained the models using the tumor grade information to establish specialized machine learning models for grade classification. Results showed that our methods accurately distinguished the images of low-to-moderate-grade cancer from those of high-grade cancer (Fig. 2a; AlexNet AUC = 0.760 ± 0.082; GoogLeNet AUC = 0.810 ± 0.067; VGGNet AUC = 0.812 ± 0.088). Grad-CAM demonstrated that the convolutional neural networks attended to the cancer cell organization patterns when differentiating histopathology images of tumors of different grades (Fig. 2b and c), demonstrating that neural networks and pathologists employed similar histology patterns in the identification of cancer cell differentiation levels.

**Fig. 3:**
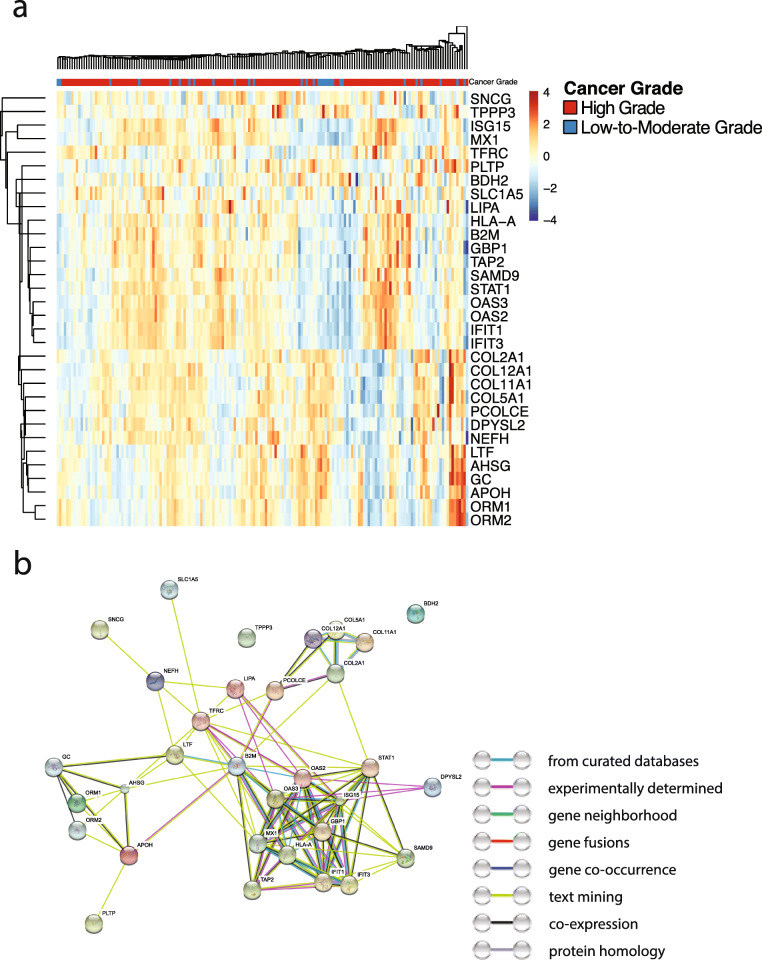
Proteomics analyses revealed the molecular profiles associated with tumor grade. **a** The expression levels of 32 proteins are associated with tumor grade. Sidebar: red indicates high-grade tumors; blue indicates low-to-moderate-grade tumors. **b** The protein-protein interaction (PPI) network of the proteins associated with tumor grade. These 32 proteins have significantly enriched PPIs (*P* < 6.66 × 10^−16^). The color of the edges shows the information source of the curated protein-protein interactions in the STRING database

We further conducted proteomics analyses using the same patient cohort to examine the molecular differences between the tumor grade groups. Our results showed that the expression levels of 32 proteins are significantly associated with the observed tumor grade under the microscope. Figure 3a shows the expression heatmap of the proteins associated with tumor grade. Gene Ontology (GO) enrichment analyses revealed that these proteins are enriched in the immune reaction and catabolic processes (Supplemental Table 1). For instance, type I interferon, cytokine-mediated, and interferon-gamma-mediated signaling pathways are among the most enriched biological processes. Collagen catabolic processes and extracellular matrix (ECM) disassembly processes are also highly enriched. KEGG pathway analyses confirmed the relevance of protein digestion, ECM-receptor interaction, and immune-related pathways to the differentiation levels of serous ovarian carcinoma (Supplemental Table 2). The enrichments of ECM-related molecular processes are consistent with the observation that cancer cell organization patterns received high weights in the grad-CAM analyses. The proteins related to tumor grade possess significant (enrichment *P* value = 6.66 × 10^−16^) protein-protein interactions (PPI; Fig. 3b). Differential analyses of the RNA-seq data identified 12 mRNA transcripts whose expression levels are associated with tumor grade (Supplemental Figure 1A; Benjamini-Hochberg corrected *P* < 0.05). Interestingly, many of these differentially expressed transcripts are non-coding RNAs (such as MIR199A1 and MIR3681), suggesting a role of post-transcriptional regulations in determining the levels of tumor differentiation. We further conducted machine learning analyses that employed proteomics and RNA-seq data to predict tumor grade. Results showed that these molecular profiles only have a weak predictive value for tumor grade (AUC < 0.6; Supplemental Figure 1B), which suggested the difficulty in tumor grade prediction using molecular data and supported the use of pathology evaluation for grade assessment.

### Convolutional neural networks identified the transcriptomic subtypes of ovarian cancer patients

The gene expression landscape of serous ovarian carcinoma samples varies across patients. Previous studies proposed four transcriptomic subtypes related to the dysregulation of genes and patient prognoses [27]. Here, we trained a convolutional neural network to connect histopathology images with the transcriptomic subtypes. We employed the VGGNet-based neural network architecture as our base model due to its reliable performance in the previous tasks, and we fine-tuned the neural connection weights using the images and transcriptomic subtype annotations to establish a specialized subtype prediction model. Results showed that the histopathology image features extracted by the model are significantly associated with the four transcriptomic subtypes (Kruskal-Wallis test *P* value < 0.0001 in PC1, *P* value = 0.0001 in PC2), indicating a cogent relation between histologic morphology and the molecular patterns underpinning the subtypes (Supplemental Figure 2A).

Due to the fact that many samples received moderate-to-high scores in more than one subtype, the definition of transcriptomic subtypes is not clear-cut. To further examine the correlations between transcriptomic subtype scores and histopathology patterns, we computed the correlation coefficients between transcriptomics-based subtype scores and the histopathology-predicted subtype scores. Results showed that there are moderate correlations between the transcriptomics-based and the histopathology-predicted scores (Spearman’s correlation: 0.235 for differentiated; 0.328 for immunoreactive; 0.576 for mesenchymal; and 0.111 for proliferative subtypes; Supplemental Figure 2B). Correlation testing showed that the associations are statistically significant in the differentiated, immunoreactive, and mesenchymal subtypes (*P* = 0.031 for differentiated; *P* = 0.002 for immunoreactive; *P* < 0.001 for mesenchymal subtypes).

### Convolutional neural networks predicted the PFI of ovarian cancer patients

**Fig. 4:**
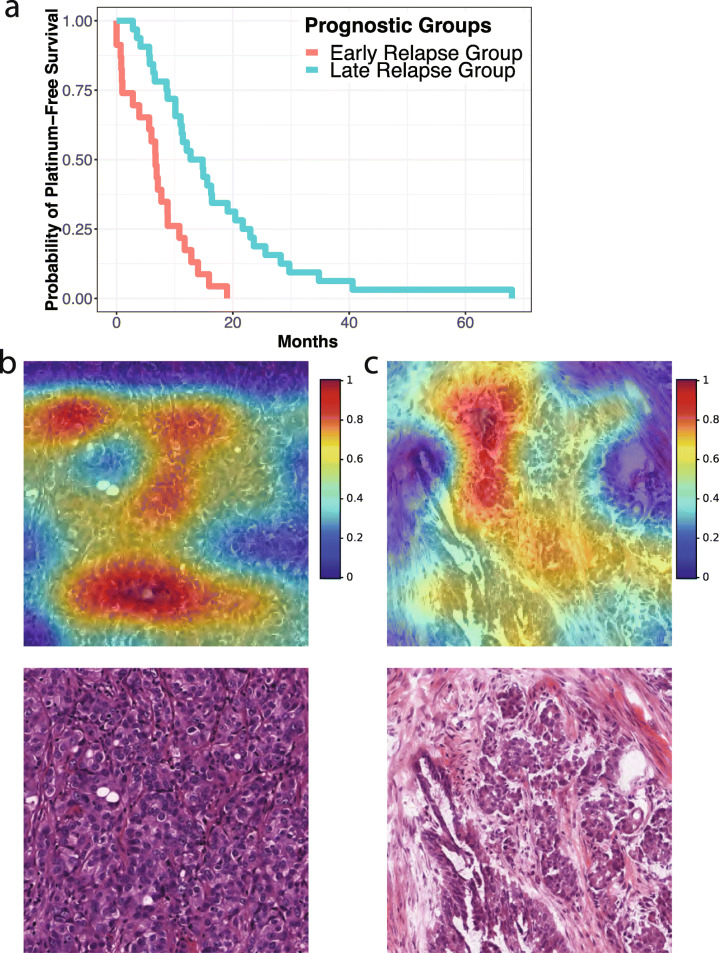
Convolutional neural networks predicted the platinum-based chemotherapy response of patients with serous ovarian carcinoma. **a** Convolutional neural networks stratified serous ovarian carcinoma patients with different platinum-based chemotherapy response (log-rank test *P* = 0.003). Kaplan-Meier curves of the image-based stratification in the test cases are shown. **b** The gradient-weighted class activation map (grad-CAM) of a histopathology image of a serous ovarian carcinoma patient with short platinum-free interval (PFI) and the original hematoxylin-and-eosin-stained histopathology image. **c** The grad-CAM of a histopathology image of a serous ovarian carcinoma patient with long PFI and the original hematoxylin-and-eosin-stained histopathology image. Grad-CAMs highlighted regions occupied by the tumor cells

Platinum-based chemotherapy is the standard treatment for advanced-stage ovarian cancer patients [10, 11]. However, it is difficult to predict which patient will respond to the treatment before administering this highly toxic chemotherapy regimen. To identify the microscopic morphological differences between patients with different chemotherapy response, we redesigned our deep learning framework to predict the PFI of each patient using their whole-slide digital histopathology images. We designed a neural network using the VGGNet architecture as the backbone and replaced the last neural layer with a regression node. Our data revealed that our convolutional neural network method accurately distinguished the histopathology images of patients with shorter PFI from those with longer PFI (log-rank test *P* = 0.003; Fig. 4a). To identify the histopathology patterns predictive of PFI, we employed grad-CAM to visualize the images with high prediction confidence. Results showed that the convolutional neural networks highlighted regions occupied by the cancer cells (Fig. 4b and c), indicating cancer cell morphologies are associated with patients’ PFIs. However, neither tumor grade nor stage is significantly associated with PFIs (log-rank test *P* > 0.056). These results suggested that subtle histological changes in the cancer cells, which are not captured by the standard histopathology annotations or pathological staging, are predictive of patients’ chemotherapy response.

**Fig. 5:**
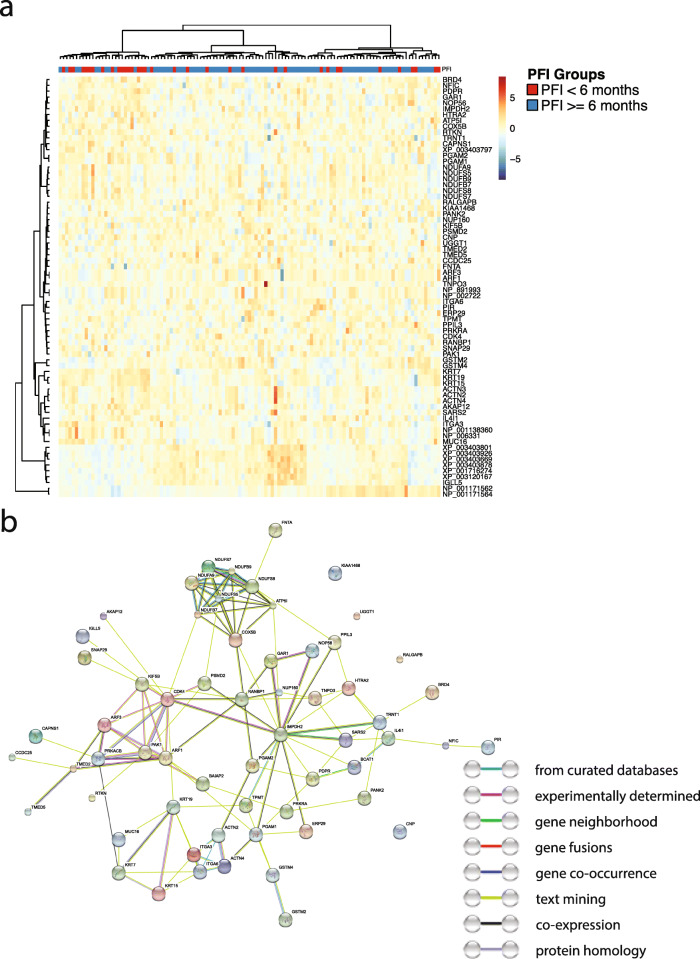
Proteomic profiles are associated with platinum-based chemotherapy response. **a** 72 proteins are significantly associated with the platinum-free interval (PFI) of serous ovarian cancer patients. **b** The interaction network of the proteins associated with PFI. Proteins associated with platinum response have significantly enriched protein-protein interactions (*P* = 7.86 × 10^−9^). The color of the edges shows the information source of the curated protein-protein interactions in the STRING database

We further conducted functional omics analyses to characterize the genes and proteins related to patients’ PFI. Proteomics analysis revealed that the expression levels of 72 proteins (including protein isoforms) are significantly correlated with patients’ PFI (Fig. 5a; all proteins shown in the figure have Benjamini-Hochberg corrected *P* value < 0.05). These proteins form a tight protein-protein interaction network (Fig. 5b; protein-protein interaction enrichment *P* value = 7.86 × 10^−9^) and are significantly enriched in purine ribonucleoside metabolic processes, ATP metabolic process, and respiratory electron transport chain (Supplemental Table 3). KEGG pathway analyses revealed significant enrichment in oxidative phosphorylation, actin regulation, and metabolic pathways (Supplemental Table 4). These molecular processes are involved in cell proliferation and cellular energetics, which are well-known hallmarks of cancer cells. Thus, these results are consistent with our findings that cancer cell histopathology, rather than the morphology of tumor stroma or inflammatory cell infiltration, is predictive of patients’ response to platinum-based chemotherapy. To investigate the extent of post-transcriptional regulations involved in patients’ platinum response, we conducted differential gene expression analyses using the RNA-seq data, which revealed 1148 mRNA transcripts significantly associated with platinum response (Supplemental Figure 3A; Benjamini-Hochberg corrected *P* value < 0.05). However, the vast majority (99.8%) of the differentially expressed genes do not have significant differential expressions at the protein level, suggesting substantial post-transcriptional regulations of the identified transcripts. In addition, machine learning analyses showed that cancer proteomic profiles possess weak signals for predicting the platinum response groups (AUC = 0.638 ± 0.014), and the predictive power of the RNA-seq data is even weaker (AUC = 0.519 ± 0.003; Supplemental Figure 3B), indicating the difficulty of platinum response prediction. Taken together, results from our functional omics analyses indicated the potential roles of cell proliferation and deregulating cellular energetics in the development of chemotherapy resistance among serous ovarian adenocarcinoma patients.

### Trained models and software dependencies

To enhance the reproducibility of our results, we released our trained machine learning models as well as our algorithms for the associated transcriptomics and proteomics analyses at https://github.com/khyu/ovarian_ca/. The GitHub repository also contains the software dependencies required to train and test the neural network models, and we provided detailed instructions on installing the required packages and the usage of our models.

## Discussion

This is the first study that associates quantitative histopathology of serous ovarian carcinoma with patients’ platinum-based chemotherapy response. In our analyses, we first demonstrated the utility of convolutional neural networks in identifying tumor cells, classifying tumor grades and transcriptomic subtypes, and we leveraged the developed machine learning platform to connect histopathology morphology with individual patients’ PFIs. We complemented the image-based predictions with functional omics analyses to further delineate the molecular processes underpinning tumor cell differentiation and treatment response. The identified correlations between histopathology patterns and chemotherapy response could facilitate treatment selection and prognosis prediction for ovarian cancer patients.

We successfully discovered morphological patterns associated with patients’ response to platinum-based chemotherapy using deep learning approaches and provide molecular explanations of the identified associations. The prediction of chemotherapy response is crucial, and many research groups have proposed biochemical and proteomic biomarkers that predict individual patient’s response [40–42]. However, the previously proposed biomarkers required additional profiling of the serum or tumor tissue, and none of them are routinely used in the clinical settings thus far. In this analysis, we identified histopathology patterns associated with platinum response and characterized the functional omics profiles and biological pathways underpinning the differential response to platinum-based chemotherapy. For instance, we demonstrated that proteins involved with purine metabolic processes and respiratory electron transport chain are significantly associated with patients’ platinum response. These molecular insights shed light on the biological pathways related to the development of platinum resistance. As the treatment strategies for ovarian cancer patients evolve, researchers can apply our computational approaches to identify the morphological and molecular signals indicative of response to newer treatments, such as neoadjuvant chemotherapy or immunotherapy.

Since histopathology evaluation is routinely used in the diagnosis of serous ovarian cancer [4], computer vision analysis on the collected histopathology slides does not require additional laboratory measurement of the tumor sample, such as sequencing or biochemical profiling [25]. Our results indicated that the morphological patterns of tumor tissue may contain previously overlooked signals related to molecular subtypes and clinical prognoses. The differential expression analyses further pointed to the molecular processes underpinning tumor grade and platinum response, such as ECM-receptor interactions in tumor grade and oxidative phosphorylation in platinum response, which suggested the biological mechanisms leading to the divergent microscopic tumor phenotypes of ovarian cancer patients. In addition, we found that the differentially expressed mRNA transcripts and proteins are not the same, although they are often involved in similar biological pathways. These results suggested post-transcriptional regulations play an important role in the molecular processes associated with cancer cell differentiation and chemotherapy response.

We further compared the performance of different neural network architectures in our classification tasks. Results showed that VGGNet-based models attained the best performance overall, closely followed by GoogLeNet, while AlexNet generally had the lowest AUC. VGGNet is an award-winning convolutional neural network known for its symmetrical design and high performance in image classification and object localization tasks [30]. It has the largest number of parameters among the model architectures we evaluated and used L2 regularization to reduce overfitting [30]. GoogLeNet has a similar number of neural layers but contains much fewer parameters [29], making it faster to train and evaluate. AlexNet has the fewest number of neural layers (5 convolutional layers and 3 fully connected layers) [28], which permits fast prototyping but suffers from limited performance. Recent studies showed that although very deep convolutional neural network models may attain better performance than VGGNet when trained on millions of images [43], their performance varied in smaller datasets [44]. Thus, VGGNet may be a reasonable starting point for biomedical datasets, which generally have a relatively small sample size. Future studies on automated model architecture search and optimization [45] may further improve the performance of prediction models trained with limited amounts of data.

It is worth noting that modest inter-rater agreement (*κ* = 0.24–0.58) in grade classification among pathologists has been reported for ovarian carcinoma [8], which contributes to the lower accuracy in machine learning models trained on the manually labeled grade annotations. Further studies on tumor classification based on objective clinical outcomes have the potential for better informing treatment selection. In addition, our study focused on serous ovarian carcinoma and did not include samples of other types of epithelial ovarian carcinoma, germ cell tumor, or distant metastases of cancers arising from other organs. Future works include extending our machine learning approaches to other rarer types of ovarian cancer as well as all cancer types. Another limitation of this study is that the treatment strategy, molecular subtypes [46], and grading guidelines evolve over time, which may render any developed model using retrospective data obsolete. Nonetheless, here, we propose a flexible machine learning training process that could accommodate categorical and continuous clinical outcomes of interest, and we can aptly retrain the diagnostic and prognostic models with histopathology images and their updated clinical labels.

## Conclusions

Our study showed that convolutional neural networks accurately predicted the cancerous regions, grade, transcriptomic subtypes, and chemotherapy response of serous ovarian carcinoma patients. Our machine learning-based approach is extensible to other tumor types and treatment modalities.

## Supplementary information

**Additional file 1: Figure S1.** Relations between tumor grade and functional omics profiles. (A) Transcriptomics analysis uncovered the 12 transcripts whose expression levels are associated with tumor grade. Sidebar: red indicates high-grade tumors; blue indicates low-to-moderate-grade tumors. (B) Proteomics and RNA-seq data have weak predictive power for tumor grade. Cross-validation AUC using proteomics data = 0.566 ± 0.016. Cross-validation AUC using RNA-seq data = 0.516 ± 0.005. **Figure S2.** Convolutional neural networks associated histopathology image patterns with the transcriptomic subtypes of serous ovarian carcinoma. (A) Features extracted by a convolutional neural network (16-layer VGGNet) are associated with transcriptomic subtypes (Kruskal-Wallis test *P* value < 0.0001 in PC1, *P* value = 0.0001 in PC2). Triangular dots represent the mean PC1 and PC2 of the four subtypes. (B) The histopathology-predicted subtype scores are moderately correlated with the subtype scores defined by the transcriptomics data (Spearman’s correlation: 0.235 for differentiated; 0.328 for immunoreactive; 0.576 for mesenchymal; and 0.111 for proliferative subtypes). The red line in each figure panel shows the regression line of the RNA-seq-defined transcriptomic subtype scores and the histopathology-predicted scores. **Figure S3.** Relations between platinum response and functional omics profiles. (A) Transcriptomic profiles of 1148 transcripts are significantly associated with the PFI of serous ovarian cancer patients. (B) Proteomics and RNA-seq data have weak predictive power for platinum response groups. Cross-validation AUC using proteomics data = 0.638 ± 0.014. Cross-validation AUC using RNA-seq data = 0.519 ± 0.003.

**Additional file 2: Table S1.** Gene Ontology (GO) enrichment analysis results of proteins associated with the grade of serous ovarian adenocarcinoma patients. **Table S2.** KEGG pathway enrichment analysis results of proteins associated with the grade of serous ovarian adenocarcinoma patients. **Table S3.** Gene Ontology (GO) enrichment analysis results of proteins associated with platinum-free interval of serous ovarian adenocarcinoma patients. **Table S4.** KEGG pathway enrichment analysis results of proteins associated with platinum-free interval of serous ovarian adenocarcinoma patients.

## Data Availability

Our machine learning models and algorithms for the associated transcriptomics and proteomics analyses are available at https://github.com/khyu/ovarian_ca/. The datasets analyzed during the current study are available from the National Cancer Institute Genomic Data Commons (https://gdc.cancer.gov/) and the Clinical Proteomic Tumor Analysis Consortium Data Coordinating Center (https://proteomics.cancer.gov/programs/cptac).
