# Supplementary figures and images for: Deciphering serous ovarian carcinoma histopathology and platinum response by convolutional neural networks
Source: BMC Med. 2020 Aug 18;18:236. doi: 10.1186/s12916-020-01684-w (PMC7433108; doi:10.1186/s12916-020-01684-w)

Supplemental Figure 1

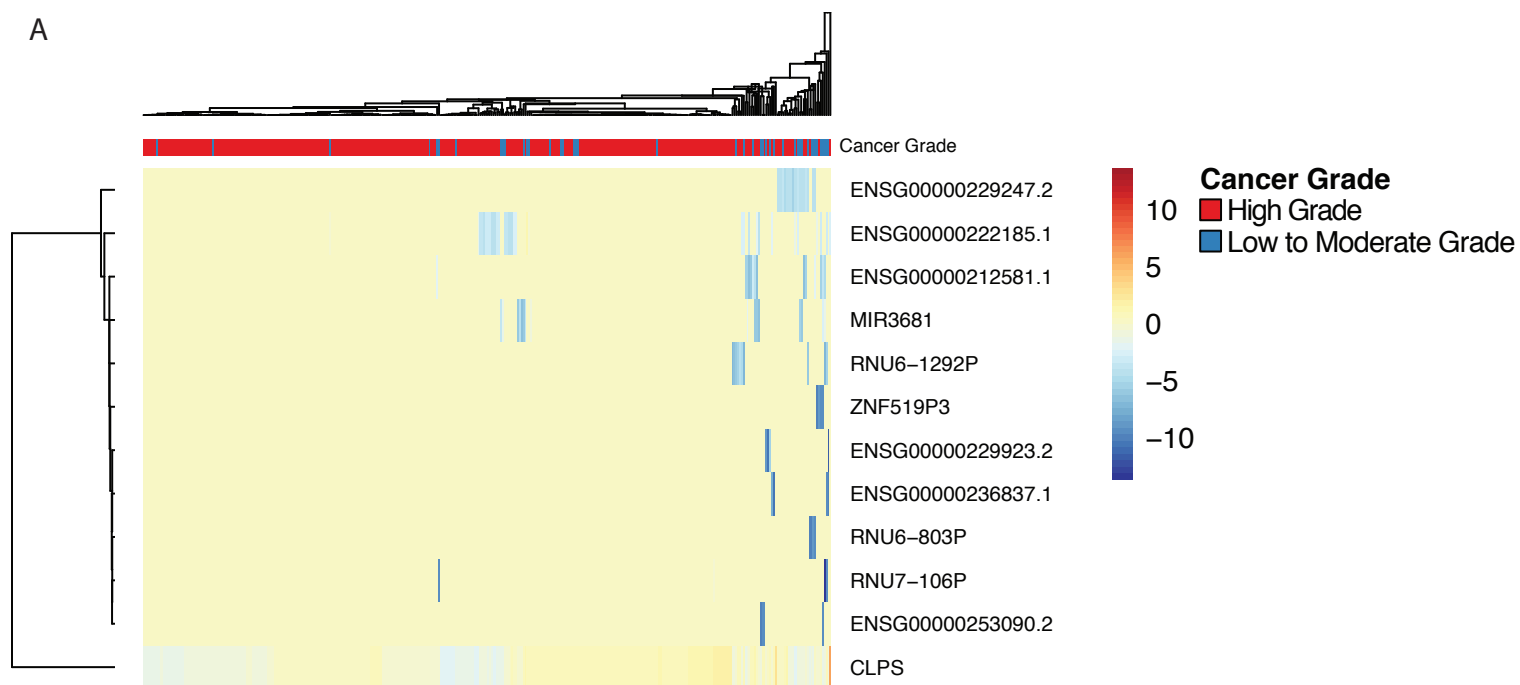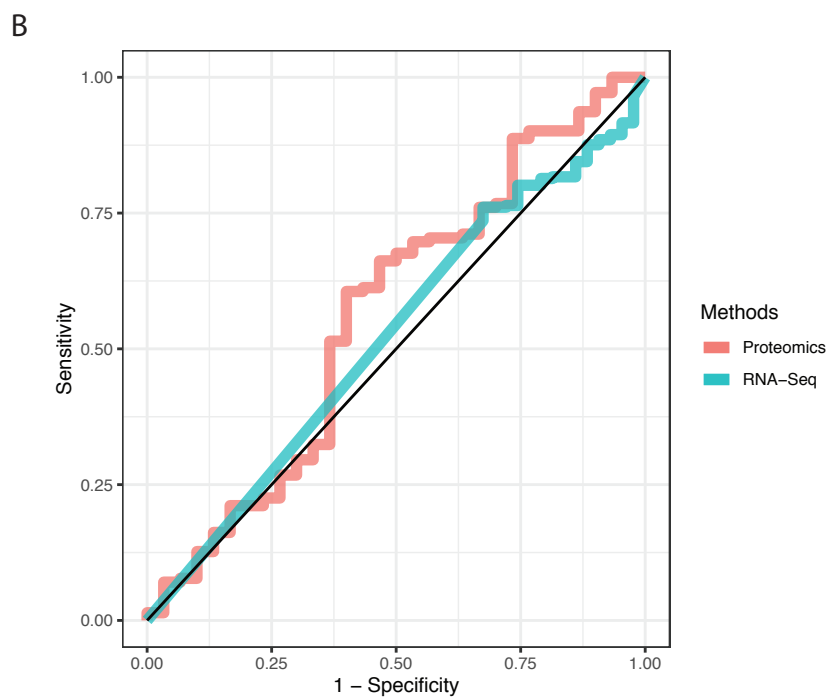

Supplemental Figure 2

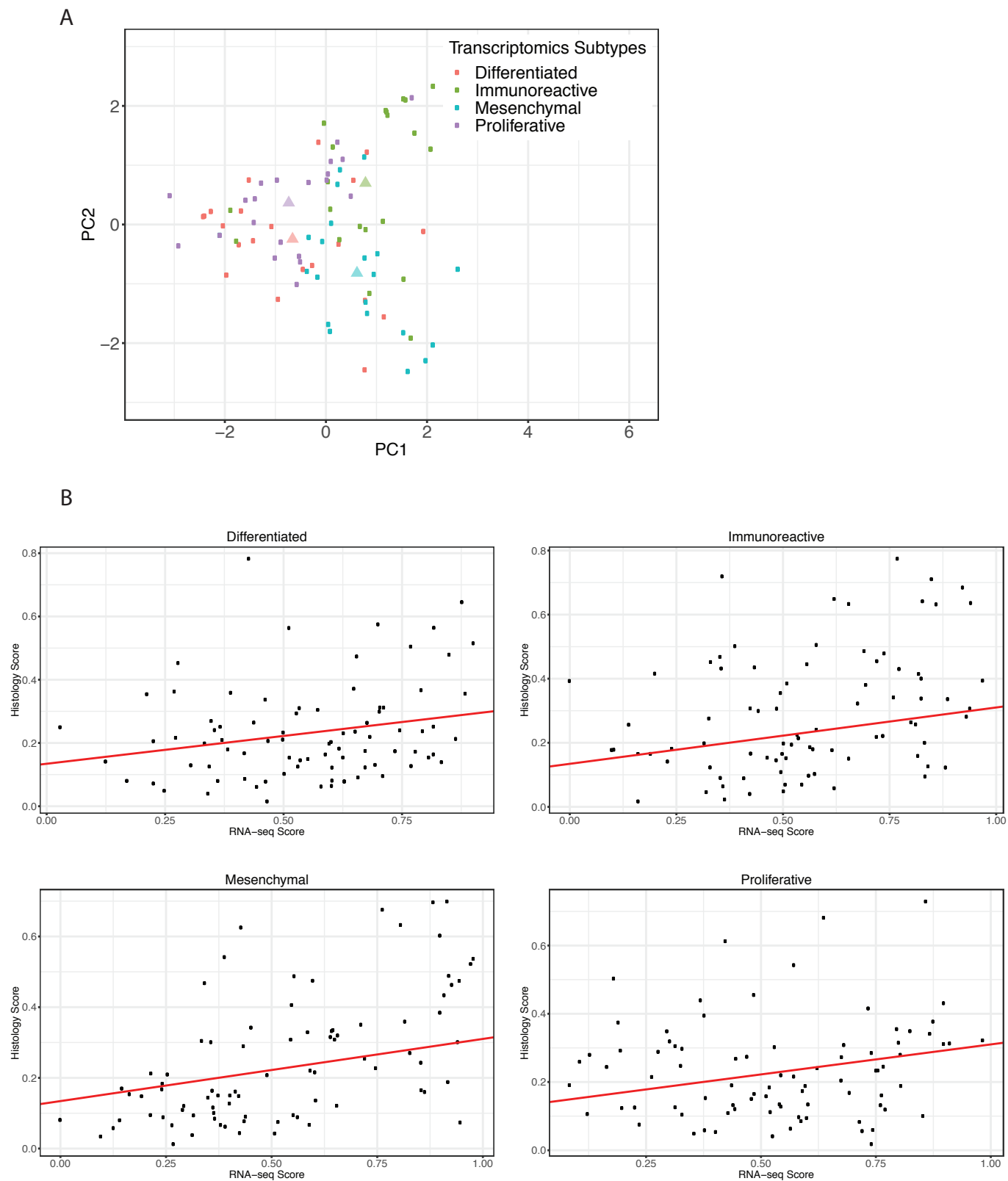

Supplemental Figure 3

A

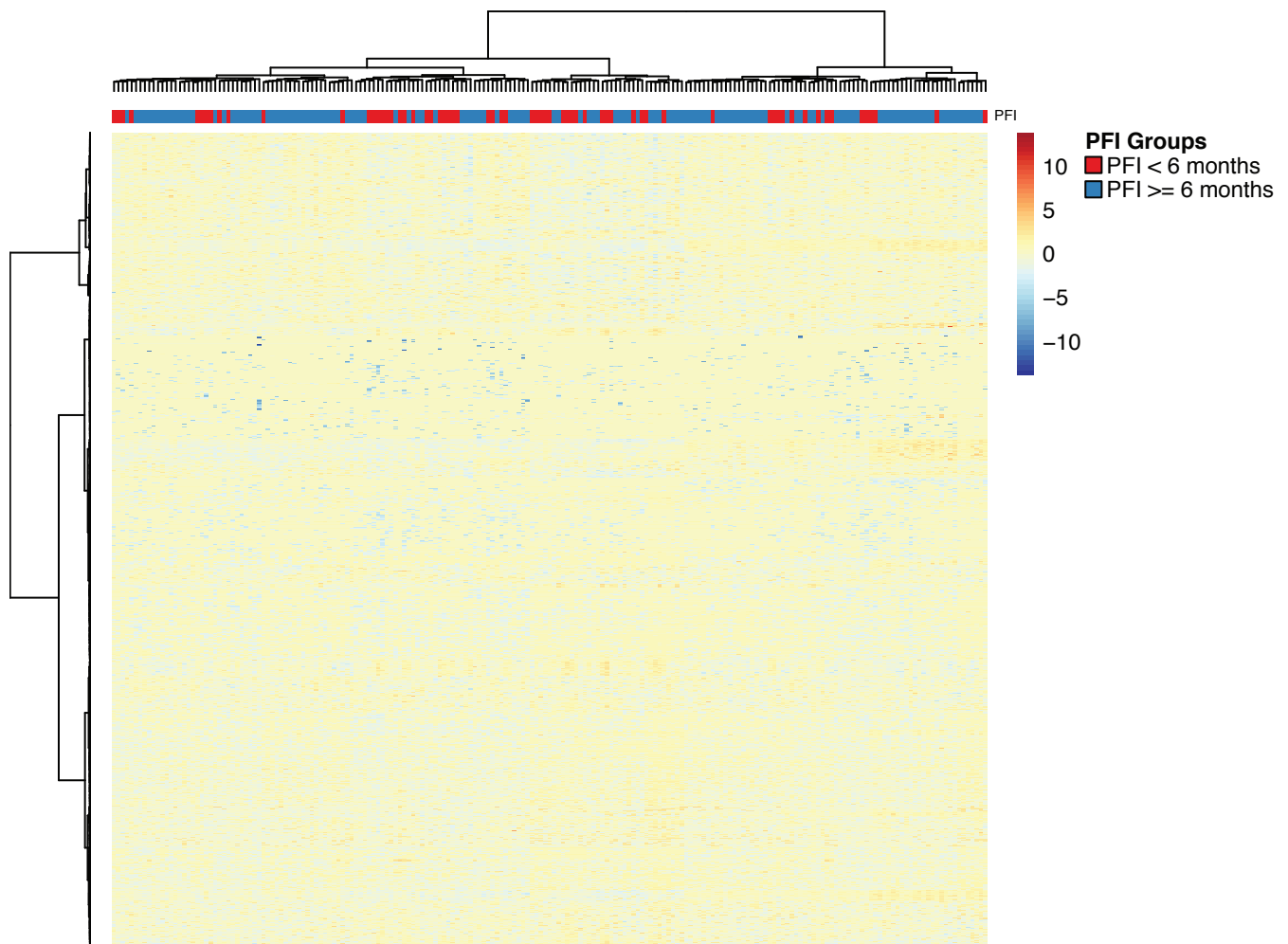

B

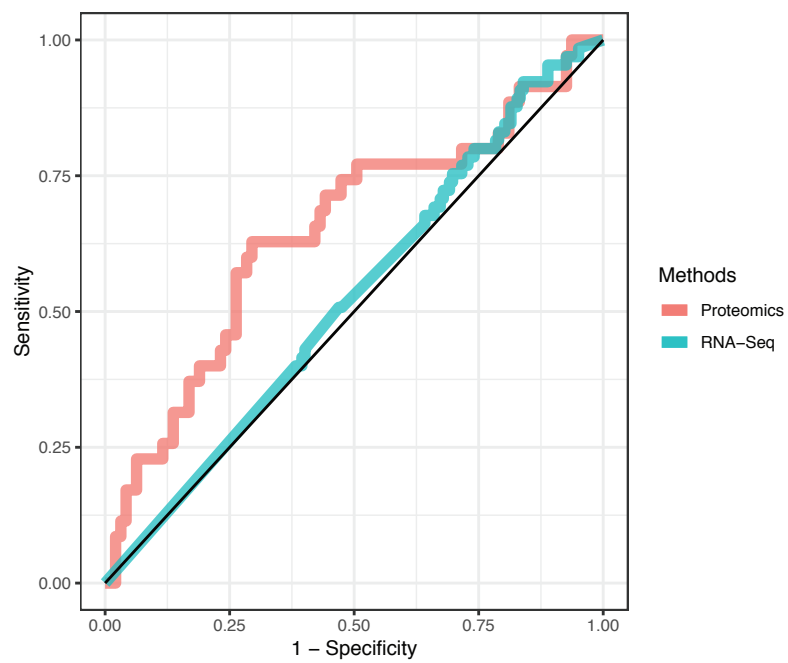

Supplement: Supplementary file 1 — Additional file 1: Figure S1. Relations between tumor grade and functional omics profiles. (A) Transcriptomics analysis uncovered the 12 transcripts whose expression levels are associated with tumor grade. Sidebar: red indicates high-grade tumors; blue indicates low-to-moderate-grade tumors. (B) Proteomics and RNA-seq data have weak predictive power for tumor grade. Cross-validation AUC using proteomics data = 0.566 ± 0.016. Cross-validation AUC using RNA-seq data = 0.516 ± 0.005. Figure S2. Convolutional neural networks associated histopathology image patterns with the transcriptomic subtypes of serous ovarian carcinoma. (A) Features extracted by a convolutional neural network (16-layer VGGNet) are associated with transcriptomic subtypes (Kruskal-Wallis test P value < 0.0001 in PC1, P value = 0.0001 in PC2). Triangular dots represent the mean PC1 and PC2 of the four subtypes. (B) The histopathology-predicted subtype scores are moderately correlated with the subtype scores defined by the transcriptomics data (Spearman’s correlation: 0.235 for differentiated; 0.328 for immunoreactive; 0.576 for mesenchymal; and 0.111 for proliferative subtypes). The red line in each figure panel shows the regression line of the RNA-seq-defined transcriptomic subtype scores and the histopathology-predicted scores. Figure S3. Relations between platinum response and functional omics profiles. (A) Transcriptomic profiles of 1148 transcripts are significantly associated with the PFI of serous ovarian cancer patients. (B) Proteomics and RNA-seq data have weak predictive power for platinum response groups. Cross-validation AUC using proteomics data = 0.638 ± 0.014. Cross-validation AUC using RNA-seq data = 0.519 ± 0.003. [file 12916_2020_1684_MOESM1_ESM.pdf]
