# Supplementary material for: Deciphering serous ovarian carcinoma histopathology and platinum response by convolutional neural networks
Source: BMC Med. 2020 Aug 18;18:236. doi: 10.1186/s12916-020-01684-w (PMC7433108; doi:10.1186/s12916-020-01684-w)
Supplement: Supplementary file 2 — Additional file 2: Table S1. Gene Ontology (GO) enrichment analysis results of proteins associated with the grade of serous ovarian adenocarcinoma patients. Table S2. KEGG pathway enrichment analysis results of proteins associated with the grade of serous ovarian adenocarcinoma patients. Table S3. Gene Ontology (GO) enrichment analysis results of proteins associated with platinum-free interval of serous ovarian adenocarcinoma patients. Table S4. KEGG pathway enrichment analysis results of proteins associated with platinum-free interval of serous ovarian adenocarcinoma patients. [file 12916_2020_1684_MOESM2_ESM.pdf]

Supplemental Table 1. Gene Ontology (GO) enrichment analysis results of proteins associated with the grade of serous ovarian adenocarcinoma patients.

Biological Processes			
**Pathway ID**	**Description**	**Gene Count**	**False Discovery Rate**
GO.0060337	type I interferon signaling pathway	8	3.90E-10
GO.0071357	cellular response to type I interferon	8	3.90E-10
GO.0006952	defense response	16	4.31E-08
GO.0051607	defense response to virus	8	1.83E-07
GO.0019221	cytokine-mediated signaling pathway	10	2.72E-07
GO.0060333	interferon-gamma-mediated signaling pathway	6	1.12E-06
GO.0009605	response to external stimulus	16	1.47E-06
GO.0098542	defense response to other organism	9	2.06E-06
GO.0048525	negative regulation of viral process	6	2.87E-06
GO.0071346	cellular response to interferon-gamma	6	5.07E-06
GO.0045071	negative regulation of viral genome replication	5	7.45E-06
GO.0006950	response to stress	19	1.08E-05
GO.0051707	response to other organism	10	1.94E-05
GO.0045087	innate immune response	11	2.97E-05
GO.0071345	cellular response to cytokine stimulus	9	3.01E-05
GO.0030199	collagen fibril organization	4	0.000179
GO.0006953	acute-phase response	4	0.000236
GO.0030574	collagen catabolic process	4	0.00176
GO.0043900	regulation of multi-organism process	7	0.00176
GO.0051704	multi-organism process	13	0.00178
GO.0055072	iron ion homeostasis	4	0.00413
GO.0044403	symbiosis, encompassing mutualism through parasitism	8	0.00442
GO.0010033	response to organic substance	13	0.0045
GO.0022617	extracellular matrix disassembly	4	0.00853
GO.0071310	cellular response to organic substance	11	0.00891
GO.0070887	cellular response to chemical stimulus	12	0.0112

GO.0002480	antigen processing and presentation of exogenous peptide antigen via MHC class I, TAP-independent	2	0.0139
GO.0035989	tendon development	2	0.0139
GO.0002376	immune system process	11	0.0148
GO.0051241	negative regulation of multicellular organismal process	8	0.0148
GO.0016032	viral process	7	0.0166
GO.0046719	regulation by virus of viral protein levels in host cell	2	0.0216
**Molecular Function**			
**Pathway ID**	**Description**	**Gene Count**	**False Discovery Rate**
GO.0001948	glycoprotein binding	4	0.00603
GO.0005201	extracellular matrix structural constituent	4	0.00603
GO.0001730	2 -5 -oligoadenylate synthetase activity	2	0.00974
GO.0030020	extracellular matrix structural constituent conferring tensile strength	2	0.0243
**Cellular Components**			
**Pathway ID**	**Description**	**Gene Count**	**False Discovery Rate**
GO.0005576	extracellular region	22	5.56E-06
GO.0044421	extracellular region part	20	8.16E-06
GO.0070062	extracellular exosome	17	1.67E-05
GO.0005615	extracellular space	12	2.62E-05
GO.0005583	fibrillar collagen trimer	3	0.000124
GO.0072562	blood microparticle	5	0.000124
GO.0098643	banded collagen fibril	3	0.000124
GO.0005581	collagen trimer	4	0.00112
GO.0005788	endoplasmic reticulum lumen	5	0.00115
GO.0042612	MHC class I protein complex	2	0.00507
GO.0030424	axon	5	0.0176
GO.0045335	phagocytic vesicle	3	0.0195
GO.0048471	perinuclear region of cytoplasm	6	0.0238
GO.0043227	membrane-bounded organelle	26	0.0314

Supplemental Table 2. KEGG pathway enrichment analysis results of proteins associated with the grade of serous ovarian adenocarcinoma patients.

Pathway ID	Description	Gene Count	False Discovery Rate
4974	Protein digestion and absorption	5	5.48E-05
5168	Herpes simplex infection	4	0.0217
4512	ECM-receptor interaction	3	0.0301
5146	Amoebiasis	3	0.0408
4611	Platelet activation	3	0.0436
5160	Hepatitis C	3	0.0436
5162	Measles	3	0.0436

Supplemental Table 3. Gene Ontology (GO) enrichment analysis results of proteins associated with platinum-free interval of serous ovarian adenocarcinoma patients.

**Biological Process**			
**Pathway ID**	**Description**	**Gene Count**	**False Discovery Rate**
GO.0044281	small molecule metabolic process	26	1.47E-07
GO.0009167	purine ribonucleoside monophosphate metabolic process	10	6.04E-07
GO.0046128	purine ribonucleoside metabolic process	11	6.04E-07
GO.0009117	nucleotide metabolic process	13	6.59E-07
GO.0009150	purine ribonucleotide metabolic process	11	8.95E-07
GO.0006091	generation of precursor metabolites and energy	12	1.06E-06
GO.0046034	ATP metabolic process	9	1.06E-06
GO.0045333	cellular respiration	9	1.20E-06
GO.0019637	organophosphate metabolic process	15	1.77E-06
GO.0022904	respiratory electron transport chain	8	1.83E-06
GO.0006120	mitochondrial electron transport, NADH to ubiquinone	6	2.30E-06
GO.0055114	oxidation-reduction process	16	3.35E-06
GO.0006796	phosphate-containing compound metabolic process	20	1.12E-05
GO.0042775	mitochondrial ATP synthesis coupled electron transport	6	1.12E-05
GO.0015980	energy derivation by oxidation of organic compounds	10	1.19E-05
GO.0044710	single-organism metabolic process	30	1.62E-05
GO.0006119	oxidative phosphorylation	6	1.93E-05
GO.1901564	organonitrogen compound metabolic process	17	0.000295
GO.0016310	phosphorylation	14	0.000338
GO.0034641	cellular nitrogen compound metabolic process	31	0.000539
GO.0071704	organic substance metabolic process	41	0.000754
GO.1901135	carbohydrate derivative metabolic process	13	0.000913
GO.1901360	organic cyclic compound metabolic process	29	0.00189

GO.0006725	cellular aromatic compound metabolic process	28	0.00233
GO.0044238	primary metabolic process	39	0.00377
GO.0032981	mitochondrial respiratory chain complex I assembly	3	0.00561
GO.0044237	cellular metabolic process	38	0.00723
GO.0006996	organelle organization	20	0.00955
GO.0018916	nitrobenzene metabolic process	2	0.0139
GO.1904064	positive regulation of cation transmembrane transport	4	0.0147
GO.0006139	nucleobase-containing compound metabolic process	25	0.0286
GO.0044093	positive regulation of molecular function	14	0.034
GO.0007005	mitochondrion organization	7	0.0343
GO.0044711	single-organism biosynthetic process	12	0.0461
**Molecular Functions**			
**Pathway ID**	**Description**	**Gene Count**	**False Discovery Rate**
GO.0008137	NADH dehydrogenase (ubiquinone) activity	6	9.48E-06
GO.0016491	oxidoreductase activity	11	0.00173
GO.0003824	catalytic activity	30	0.00731
GO.0004082	bisphosphoglycerate mutase activity	2	0.0239
GO.0004083	bisphosphoglycerate 2-phosphatase activity	2	0.0239
**Cellular Components**			
**Pathway ID**	**Description**	**Gene Count**	**False Discovery Rate**
GO.0005747	mitochondrial respiratory chain complex I	6	3.32E-06
GO.0070062	extracellular exosome	25	8.87E-06
GO.0005746	mitochondrial respiratory chain	6	1.22E-05
GO.0043227	membrane-bounded organelle	50	6.24E-05
GO.0031988	membrane-bounded vesicle	26	6.81E-05
GO.0031967	organelle envelope	14	9.54E-05
GO.0031982	vesicle	26	0.000102
GO.0044421	extracellular region part	26	0.000182
GO.0044429	mitochondrial part	12	0.000399
GO.0098796	membrane protein complex	12	0.000607
GO.0031143	pseudopodium	3	0.000796
GO.0005576	extracellular region	27	0.00109
GO.0005740	mitochondrial envelope	10	0.00119
GO.0005743	mitochondrial inner membrane	8	0.0036

GO.0005758	mitochondrial intermembrane space	4	0.00383
GO.0048471	perinuclear region of cytoplasm	9	0.00388
GO.0031966	mitochondrial membrane	9	0.00436
GO.0005829	cytosol	21	0.00468
GO.0005925	focal adhesion	7	0.00468
GO.0043231	intracellular membrane-bounded organelle	43	0.00491
GO.0005739	mitochondrion	14	0.00561
GO.0030017	sarcomere	5	0.00772
GO.0044444	cytoplasmic part	34	0.00772
GO.1902494	catalytic complex	10	0.0103
GO.0005737	cytoplasm	41	0.0114
GO.0030018	Z disc	4	0.0114
GO.0043226	organelle	46	0.0137
GO.0044446	intracellular organelle part	34	0.0143
GO.0031674	I band	4	0.0167
GO.0032991	macromolecular complex	24	0.0194
GO.0030055	cell-substrate junction	6	0.0249
GO.0030863	cortical cytoskeleton	3	0.0323
GO.1902911	protein kinase complex	3	0.033
GO.0005732	small nucleolar ribonucleoprotein complex	2	0.0371
GO.0043234	protein complex	21	0.0411
GO.0005793	endoplasmic reticulum-Golgi intermediate compartment	3	0.0451
GO.0044422	organelle part	33	0.046

Supplemental Table 4. KEGG pathway enrichment analysis results of proteins associated with platinum-free interval of serous ovarian adenocarcinoma patients.

Pathway ID	Description	Gene Count	False Discovery Rate
5012	Parkinson's disease	8	2.31E-06
190	Oxidative phosphorylation	7	1.67E-05
4932	Non-alcoholic fatty liver disease (NAFLD)	6	0.000477
5010	Alzheimer's disease	6	0.00067
5016	Huntington's disease	6	0.00094
4810	Regulation of actin cytoskeleton	6	0.00171
5412	Arrhythmogenic right ventricular cardiomyopathy (ARVC)	4	0.00274
1100	Metabolic pathways	12	0.00496
5203	Viral carcinogenesis	5	0.00652
4510	Focal adhesion	5	0.00974
1230	Biosynthesis of amino acids	3	0.0287
4520	Adherens junction	3	0.0287
5222	Small cell lung cancer	3	0.0447
5414	Dilated cardiomyopathy	3	0.0474
